# Passive Back Support Exoskeleton Improves Range of Motion Using Flexible Beams

**DOI:** 10.3389/frobt.2018.00072

**Published:** 2018-06-21

**Authors:** Matthias B. Näf, Axel S. Koopman, Saskia Baltrusch, Carlos Rodriguez-Guerrero, Bram Vanderborght, Dirk Lefeber

**Affiliations:** ^1^Robotics and Multibody Mechanics Research Group, Department of Mechanical Engineering, Vrije Universiteit Brussel and Flanders Make, Brussels, Belgium; ^2^Amsterdam Movement Sciences, Department of Human Movement Sciences, Faculty of Behavioural and Movement Sciences, Vrije Universiteit Amsterdam, Amsterdam, Netherlands; ^3^Research and Development, Rehabilitation Centre Heliomare, Wijk aan Zee, Netherlands

**Keywords:** lower back pain, exoskeleton, range of motion, biomechanical testing, industry

## Abstract

In the EU, lower back pain affects more than 40% of the working population. Mechanical loading of the lower back has been shown to be an important risk factor. Peak mechanical load can be reduced by ergonomic interventions, the use of cranes and, more recently, by the use of exoskeletons. Despite recent advances in the development of exoskeletons for industrial applications, they are not widely adopted by industry yet. Some of the challenges, which have to be overcome are a reduced range of motion, misalignment between the human anatomy and kinematics of the exoskeleton as well as discomfort. A body of research exists on how an exoskeleton can be designed to compensate for misalignment and thereby improve comfort. However, how to design an exoskeleton that achieves a similar range of motion as a human lumbar spine of up to 60° in the sagittal plane, has not been extensively investigated. We addressed this need by developing and testing a novel passive back support exoskeleton, including a mechanism comprised of flexible beams, which run in parallel to the spine, providing a large range of motion and lowering the peak torque requirements around the lumbo-sacral (L5/S1) joint. Furthermore, we ran a pilot study to test the biomechanical (*N* = 2) and functional (*N* = 3) impact on subjects while wearing the exoskeleton. The biomechanical testing was once performed with flexible beams as a back interface and once with a rigid structure. An increase of more than 25% range of motion of the trunk in the sagittal plane was observed by using the flexible beams. The pilot functional tests, which are compared to results from a previous study with the Laevo device, suggest, that the novel exoskeleton is perceived as less hindering in almost all tested tasks.

## 1. Introduction

Lower Back Pain (LBP) and shoulder pain affects more than 40% of the working population in the EU (Eurofound, 2012). Mechanical loading has been identified as an important risk factor to develop LBP (Coenen et al., 2014). Especially, compression forces on the lumbar spine are one of the main risks, as reflected in the NIOSH standard for lifting (Waters et al., 1993).

Despite these insights, many workers are still exposed to mechanical loading: More than 40% of the workers in the EU are working in tiring and painful positions, while more than 30 % are required to lift heavy loads at least a quarter of the work time (Eurofound, 2012).

Besides the health risks for the workers, these numbers also have quite severe financial implications: Cost estimates for health expenditure, caused by lower back pain range from 116€ per capita in Belgium up to 209€ per capita in Sweden (Dagenais et al., 2008). This means, that for a relatively small country like Belgium with a population of approximately 11 million inhabitants, the total costs caused by lower back pain are as high as 1.2 billion €, which corresponds roughly to 2‰ of the gross domestic product of Belgium.

It is therefore not surprising, that cranes (Lavender et al., 2013), hoist and other means to bypass the loading of the back have been developed. However, the use of cranes and hoists and other, on site mounted means, is often infeasible due to space restrictions or not practical (Waters et al., 1994).

Therefore, more recently exoskeletons (de Looze et al., 2015) have been developed to mitigate the health risk for workers and to reduce the cost caused by injuries.

Over the last decades, several exoskeletons specifically designed to support workers have been developed: These range from exoskeletons for shoulder, lower back and leg support to exoskeletons, that support the entire body. For an extensive overview of the state of the art, the interested reader is directed to a review article of de Looze et al. (2015).

**Figure 1:**
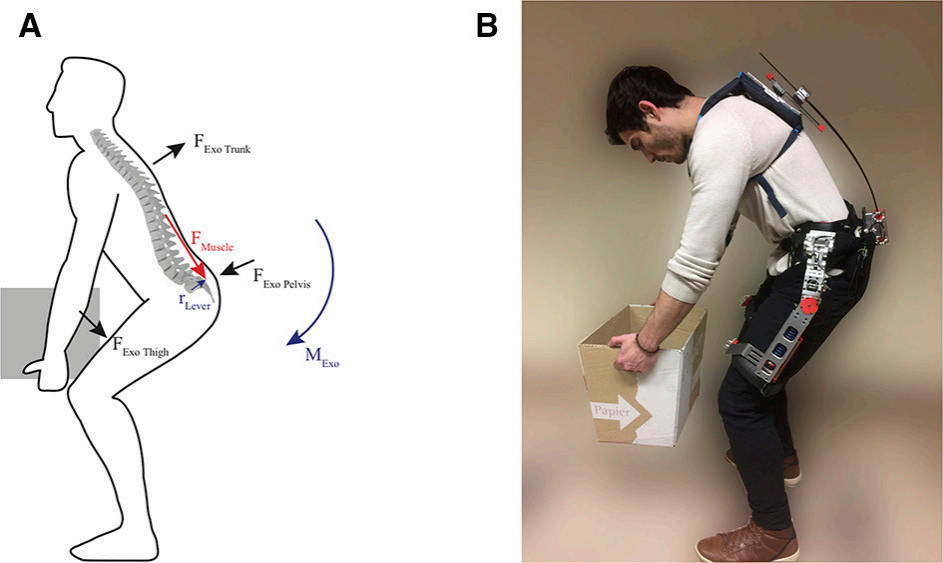
Back muscle forces and exoskeleton forces and moments **(A)**. In order to balance the gravitational forces acting on on the trunk, the back muscles (*F*_*Muscle*_) contract. Since these forces act over a small lever arm of only a few centimeters (*r*_*Lever*_), almost parallel to the spine, the spine is subject to large compression forces. Exoskeletons aim at reducing these forces, by applying forces on the trunk (*F*_*ExoTrunk*_), pelvis (*F*_*ExoPelvis*_), and thigh (*F*_*ExoThigh*_). Together they produce an extension moment (*M*_*Exo*_); Passive spexor exoskeleton **(B)**. The exoskeleton unloads the back by applying a force at the torso, pelvis, and the thighs. The user has a large range of motion, when wearing the passive exoskeleton, due to advanced misalignment compensating mechanisms like the flexible beams in parallel to the spine. Written informed consent was obtained from the participant to publish this picture.

Biomechanic considerations suggest, that the lumbo-sacral (L5/S1) region experiences peak mechanical loading in a wide range of tasks, specifically large compression forces of the spine (Coenen et al., 2014). These forces can range up to 5000 N or more when lifting a 15 kg load (Kingma et al., 2010). They are mainly due to muscle forces, needed to counteract the moment at the lower back, induced by gravitational forces on the upper body and load (See Figure 1A). Therefore, the majority of the exoskeletons and exosuits focus on reducing compression forces in the lumbo-sacral region, by lowering the muscle forces that are required for lifting or holding of a static trunk posture. In almost all designs, this is achieved either by external forces that run parallel to the human back, or moments that help extend the back. Applying these forces and moments to the torso and below the lumbo-sacral joint (L5S1) mechanically unloads the lower back.

Since the properties of these back support exoskeletons and exosuits differ significantly with the way they are constructed, they are further subdivided into two groups: rigid and soft. In this context, rigid means, that the exoskeleton structure can transmit compression as well as extension forces, whereas soft refers to, that only compression forces can be transmitted.

Classical rigid exoskeletons such as Laevo (Laevo, 2018), Robomate (Toxiri et al., 2016), Bending Non-Demand Return (BNDR) (Ulrey and Fathallah, 2013), Wearable Moment Restoring Device (WMRD) (Wehner et al., 2009), BackX (SuitX, 2018), and Back Support Muscle Suit (Muramatsu et al., 2013) have in common, that at least one single actuated joint is placed at the hip level or slightly above to allow movement in the sagittal plane. Further, a mechanical structure extends from this joint, to the trunk, and to the thigh.

**Figure 2:**
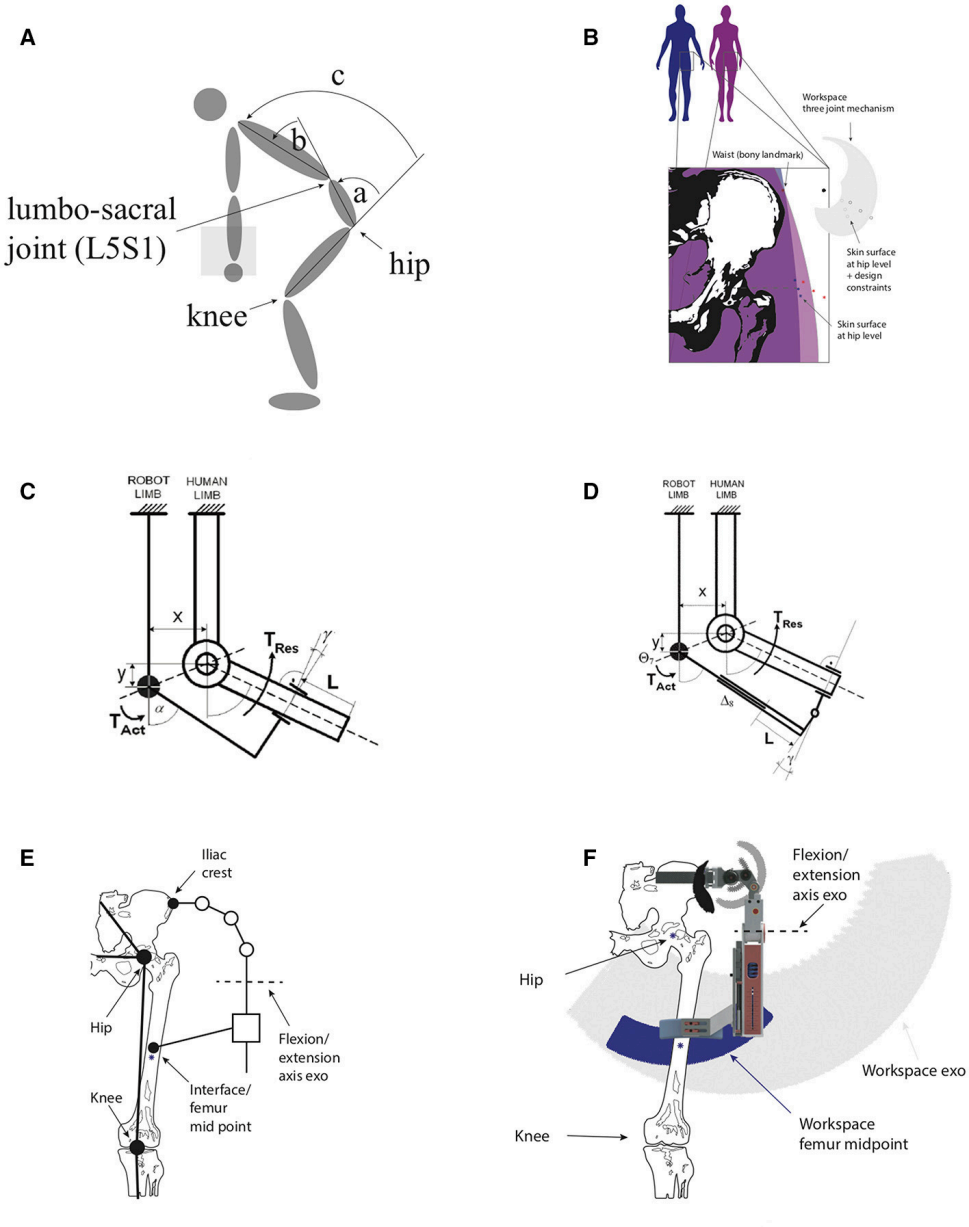
Human angle definitions **(A)** hip angle (a), lumbar angle (b), and trunk angle (c). Fitting requirements **(B)**. The relative distance between the waist and the skin surface at hip level differs significantly between man and women and within these groups. Here, the skeleton structure (adopted from Netter, 2011) is scaled to matches the 50 percentile male (blue asterisk middle). The other two blue data points indicate the 5 percentile and 95 percentile male according to Tilley (2002). The red asterisks indicate the same data points for women. Additional design constraints are accommodated by translating these data point; Two structures, that are misaligned **(C)**: The robot limb and the human limb are horizontally misaligned by a distance *x* and vertically by a distance *y*. A torque *T*_*Act*_ is applied by the robot limb, displacing it by an angle α. A resulting torque *T*_*Res*_ displaces the human limb. The initial misalignment *x* and *y* result in a displacement *L* and a rotation γ of the cuff on human limb. Figure from Schiele and van der Helm (2006); Added degrees of freedom **(D)**: no more relative motion occurs, because the misalignment is compensated by the slider Δ*s* and hinge γ. Figure from Schiele and van der Helm (2006); **(E)**: Exoskeleton together with human skeleton. See Supplementary Materials for detailed model; Workspace comparison **(F)**: the workspace of the human hip is compared to the exoskeleton workspace in the frontal plane.

However, an important aspect, which sets these devices apart, is the degree, to which the kinematic structure of the exoskeleton is designed to align with the human. Misaligned joints can produce unwanted, parasitic forces and torques of up to 230 N and 1.5 Nm, respectively (Schiele, 2009), which decrease the comfort of wearing a device. Because good alignment of an external exoskeleton structure is challenging, devices often compensate for misalignment (Junius et al., 2017a,b), i.e., instead of trying to align the exoskeleton structure with the human, a certain amount of misalignment is accepted and compensated with the exoskeleton by introducing additional degrees of freedom (see Figures 2C,D). Furthermore proper misalignment compensation prevents relative movement between the device and the user and thereby indirectly also increases comfort (Schiele and van der Helm, 2006).

The relatively big ranges of motion of the human trunk in the sagittal plane originates from the fact, that a human can flex or extend his hip joint as well as the lumbar joint(s). This leads to a certain redundancy, by which objects can be picked up. Two well-established cases include: a predominant use of the hip joint with small lumbar angles, which is commonly referred to as a squat lift, or a predominant use of the lumbar joint(s) with relatively small hip angles, commonly referred to as stoop lift (Kingma et al., 2010). These lifting styles have consequences on the torques that need to be provided at the lumbo-sacral joint: depending on lifting conditions such as object height and size, moments at the L5/S1 joint can be substantially higher in either stoop or squat lifts (Kingma et al., 2010). The only exoskeleton of the above mentioned ones, that accounts for this additional degree of freedom in the sagittal plane with an additional joint, is the Back Support Muscle Suit (Muramatsu et al., 2013). This device has, next to the hip joint, an additional joint, which is placed at the bottom of the back. Unfortunately, no further evaluation of this additional joint was found. The other exoskeletons can still have a large range of motion of the trunk. However, they do not adopt their kinematics or support with variations in lifting style.

Kinematic compatibility in the frontal and transverse planes should also be considered. The Laevo system incorporates rotating elements in the chest pad, which to some degree fulfill the function of a differential transmission. The alignment for the legs is provided through the elasticity of a belt behind the waist. In comparison, the Robomate exoskeleton features more elaborated misalignment compensation for the hip, consisting of two hinge joints and one ball joint (Toxiri et al., 2016). The same compensation mechanism is used for the trunk (Toxiri et al., 2016).

The BNDR does not include any mechanism to account for kinematic compatibility (Ulrey and Fathallah, 2013) other than the hip joints consisting of torsional springs. For the WMRD exoskeleton it is not clear, if any additional compensation mechanisms are integrated at the hip joint (Wehner et al., 2009). Two different models of the BackX exist, the model S and AC. The model S is similar in structure to the BNDR but features an additional adduction/abduction joint. The model AC, which features a rigid waist belt, also has an adduction/abduction joint and on top of that a joint at the base of the back structure to allow for lateral bending. A rotational joint at the top of the support strut enables axial rotation in the transverse pane.

Soft, suit like structures have a relatively long history in back support devices (Abdoli-Eramaki et al., 2007). The Personal Lift Augmentation Device (PLAD) was one of the pinoeers in this field (Abdoli-Eramaki et al., 2007). Like other exosuits, the forces are transmitted in tension only and no weight bearing structure parallel to the human exists.

While the lack of rigid mechanic structures greatly enhances kinematic compatibility, the range of motion can still be a challenge (Abdoli-Eramaki et al., 2007). Other devices, that use suit like structures are the Smart Suit Light (SSL) (Imamura et al., 2014), the Passive Spine Exoskeleton (Zhang et al., 2016), and the waist assist suit AB-Wear (Inose et al., 2017).

However, Inose et al. (2017) raised concerns that their waist assist suit AB-Wear, without any rigid structure, might generate unwanted compression forces. To avoid these compression forces, they updated their design to include a flat spring mechanism in the back as a load bearing structure. As the mathematical model from Abdoli-Eramaki et al. (2007) shows, suit like structures are able to reduce compression forces, as long as the force application of the suit occurs with a bigger lever arm, than that of the back muscles. However, the potential to reduce spinal compression forces is bigger, if an external, load bearing, “rigid" structure is present and thereby forces can be applied perpendicular to the back rather than tangential.

Several reasons are reported in the literature as to why back support exoskeletons and exoskeletons in general are not widely adopted in the industry yet: discomfort (Barrett and Fathallah, 2001; Abdoli-Abdoli-Eramaki et al., 2006; Bosch et al., 2016), excessive force application (Abdoli-Abdoli-Eramaki et al., 2006), loss of range of motion (Abdoli-Abdoli-Eramaki et al., 2006; Toxiri et al., 2016; Baltrusch et al., 2017), not easy to use (Barrett and Fathallah, 2001) kinematic incompatibility (Barrett and Fathallah, 2001), long donning times (Junius et al., 2017a), and lack of versatility to be used in a variety of real world settings (Baltrusch et al., 2017).

To address these needs and to develop more suitable exoskeleton solutions, the SPEXOR consortium was formed. With the goal to develop and test, first a passive, and later on, an active exoskeleton for back support (Babič et al., 2017).

This paper presents the design and preliminary testing of a passive back support exoskeleton, that allows for a large range of motion of the lumbar spine and the hip and asserts kinematic compatibility with the user (See Figure 1B). In section 2.1 the requirements which lead to the current prototype are discussed. The concept of the novel back support exoskeleton and its design are explained in section 2.2, with a focus on the elastic back support mechanisms. The mechanical implementation of the elastic back support mechanism, the misalignment compensating hip module as well as the passive torque source at the hip are described in section 2.3. Subsequently, the experimental testing of the components as well as the testing involving human subjects are elaborated in section 3. The outcomes of the component testing, biomechanical testing and functional testing are presented in section 4 and discussed thereafter in section 5.

## 2. Materials and methods

### 2.1. Requirements

An ideal back support exoskeleton can reduce the peak and cumulative loading on the back while at the same time still allow for a large range of motion. In order to be effective, a back support exoskeleton needs to connect the torso an the thighs. Due to the complexity of the hip joint and lumbar spine, many degrees of freedom need to be carefully bridged (See Figure 2A).

The kinematic compatibility and the fit of the device are tightly linked to the comfort of the user. Both characteristics are discussed in more detail in the following.

#### 2.1.1. Kinematic compatibility and fitting

In the process of designing back support exoskeletons, the degrees of freedom introduced by the lumbar spine are not always taken into account or are merged with the degrees of freedom of the hip (Wehner et al., 2009; Ulrey and Fathallah, 2013; Toxiri et al., 2016; Laevo, 2018; SuitX, 2018). Simple models of the human spine, suggest, that the lumbar spine can be modeled as one additional joint in the sagittal plane (see Figure 2A). More complex models, consist of five separate spherical joints to model the lumbar spine (Christophy et al., 2012). The angular displacement in the lumbar spine can occur in the sagittal plane by flexing or extending the spine. Lateral bending in the frontal plane and axial rotation in the transverse plane constitute the rest of the possible angular displacements. Peak values for the range of motion of the human hip, spine and trunk can be found in Table 1.

**Table 1:** Range of motion of the hip, lumbar spine and the combination of the two: trunk angle (Magee, 2006): The definitions of the flexion angles (^*^) are shown in Figure 2A.

	Flexion*	**Extension**	**Lateral bending/**		**Axial rotation/**	
			**Abduction**	**Adduction**	**Internal rotation**	**External rotation**
Hip (a)	120°	15°	−/		−/	
			50°	30°	40°	60°
Lumbar (b)	60°	35°	20 °/		18°/	
			−	−	−	−
Trunk (c)	180°	50°	70°		78°	

*Note, 33% of the trunk flexion [see Figure 2A (c)] originates from the lumbar spine [See Figure 2A (b)]*.

The relatively large amount of lumbar flexion of around 60° is not accounted for in most exoskeleton designs. This is astounding, since approximately 33% of the range of motion of the trunk flexion, comes from the lumbar spine (see Table 1).

The hip joint is commonly modeled as a a ball joint (Pons, 2008). Its degrees of freedom are comprised of flexion and extension in the sagittal plane, abduction and adduction in the frontal plane and internal external rotation in the transverse plane.

The main reason, why the degrees of freedom of the human are important, is the fact, that a misaligned exoskeleton joint can cause some unwanted forces and moments (Schiele and van der Helm, 2006). Additionally it can lead to unwanted relative movements between the device and the wearer. This problem is well-documented in Schiele and van der Helm (2006) and is common in all exoskeleton joints that are not aligned (See Figure 2C).

However, an exoskeleton joint can be misaligned without causing harm, as long as it is properly compensated. There are several ways to solve this. One that is used in a number of devices (Schiele and van der Helm, 2006; Schiele, 2009; Toxiri et al., 2016; Junius et al., 2017a), is the introduction of additional degrees of freedom (See Figure 2D).

Anthropomorphic data (Tilley, 2002) suggests that significant differences exist in hip and waist width (See Figure 2B). These differences are in the range of several centimeters. If not properly accounted for, these differences can be a source of discomfort and misalignment.

#### 2.1.2. Kinetic requirements

Due to its location at the base of the back, the peak torque in the human spine is generated a the lumbo-sacral joint (L5/S1). Bio-mechanical linked segment models (Kingma et al., 2001) suggest that peak torques of up to 254 Nm are generated around L5/S1 while lifting a load of 15.7 kg.

Especially in the design of a passive exoskeletons, any attempt to compensate for the full dynamic torque at L5/S1 would lead to an exoskeleton that hinders its user most of the time. Therefore, designers often decide to compensate for a fraction of the full dynamic torque, which ranges typically between 20 and 30 Nm for purely passive devices (Abdoli-Eramaki et al., 2007).

More complex, optimization based models indicate, that torque requirements for a passive back support exoskeleton could be as low as approximately 20 Nm (Millard et al., 2017). However, the toques of an active system for the lumbar spine and hip are significantly higher. In the optimization, the active torques were saturated to 67 Nm.

### 2.2. Concept

Based on the requirements above there were two main design goals: kinematic compatibility and the support torque in the same order of magnitude as in passive back support exoskeletons of approximately 20–30 Nm, should be transmitted. Here, first the concept for the spinal part is described, followed by the concept for the hip part.

#### 2.2.1. Spinal structure concept

Since it is challenging even for skilled people to correctly align an exoskeleton (Schorsch et al., 2014), we decided to strive for a design, where only a minimal amount of initial alignment is required. Correct alignment even with single hinge joints, such as the elbow, are a challenge (Schiele and van der Helm, 2006). Therefore, correct alignment with all five spherical joints of the lumbar spine is arduos. However, since good results can be expected by compensating for misalignment, this approach was chosen here.

**Figure 3:**
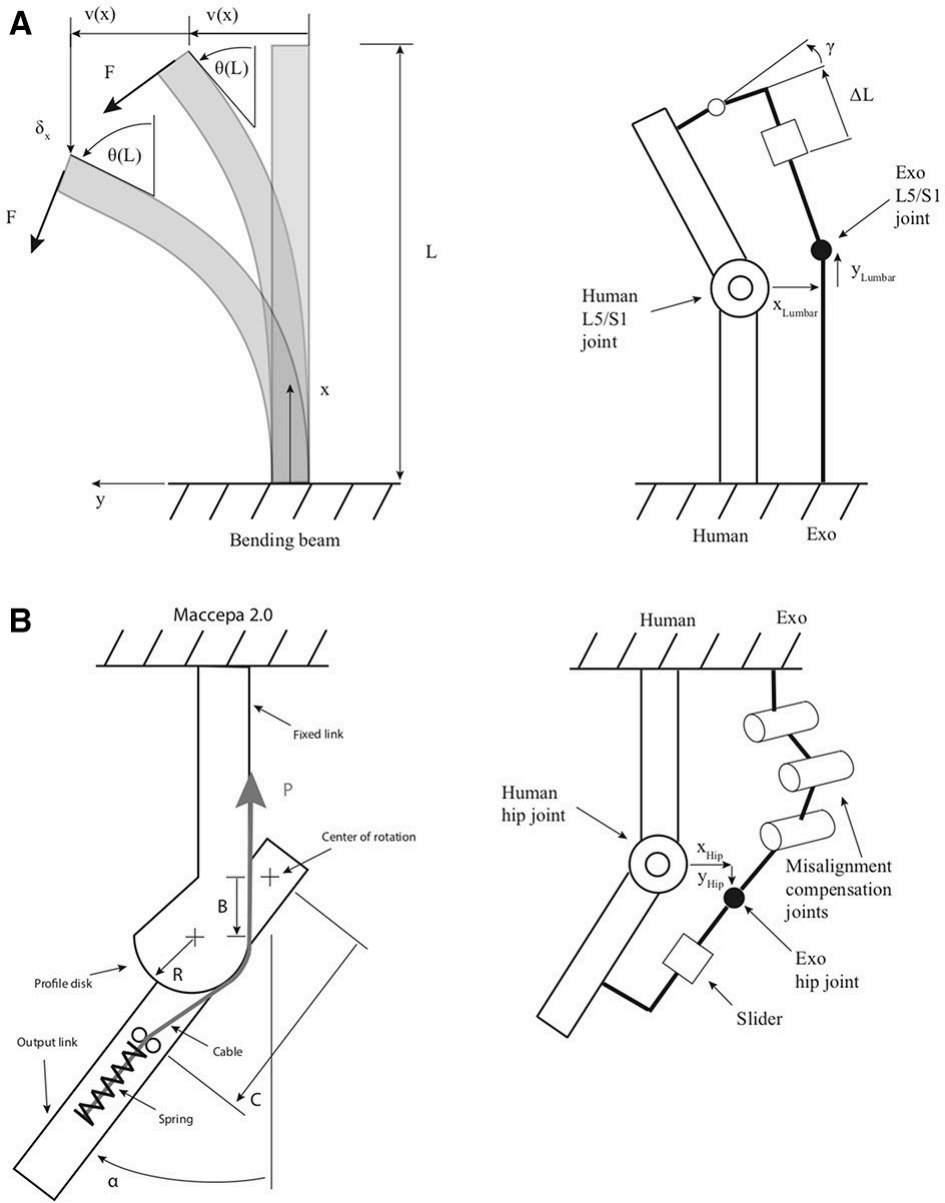
Spinal support concept **(A)**. The lumbar joint of the exoskeleton consists of a flexible beam, which serves at the same time as a torque source. On the beam of length *L* acts a force *F* at the top. This leads to a displacement *v*(*x*) which is a function of the position along the beam *x*. With increasing bending angle θ(*L*) the displacement at the top δ_*x*_ along the x-axis becomes more pronounced; The misalignment compensation mechanism for the trunk asserts, that despite a misalignment of *x*_*Lumbar*_ and *y*_*Lumbar*_ no relative movement (Δ*L*) or angular discrepancies persist (γ); Hip support concept **(B)**. The flexion extension joint is powered with a Maccepa 2.0 (Vanderborght et al., 2011), which consists of a profile disk over which a cable is tensioned. The movement of the output joint by angle α compresses a spring and thereby stores energy. The pretension *P* is altered with a worm gear. The main design parameters are an offset *B* from the center of rotation, (here) a fixed profile radius *R* and the length *C*; The misalignment compensation of the hip asserts kinematic compatibility with additional three parallel joints and a slider.

Additional degrees of freedom can be added in many forms. One that at the same time allows to store energy, is the use of flexible materials (See Figure 3A). A long and slim flexible structure can be bent in two directions and deformed under torsion. However, even to compensate for flexion and extension in the sagittal plane, at least two additional compensating degrees of freedom are required (Schiele and van der Helm, 2006). One to account for the length change, and one for correct alignment with the back. The same holds for the lateral bending and axial rotation.

The choice of the position of the additional degrees of freedom used to provide compensation is relatively arbitrary, but attention should be payed to singular positions of the used mechanism (Schiele, 2009). However, the choice of a mechanism can have practical implications (Junius et al., 2017a). Choosing a center of rotation of an exoskeleton joint, to be located relatively far away from the corresponding human joint, will require a big compensatory effort, compared to almost coinciding joints.

**Figure 4:**
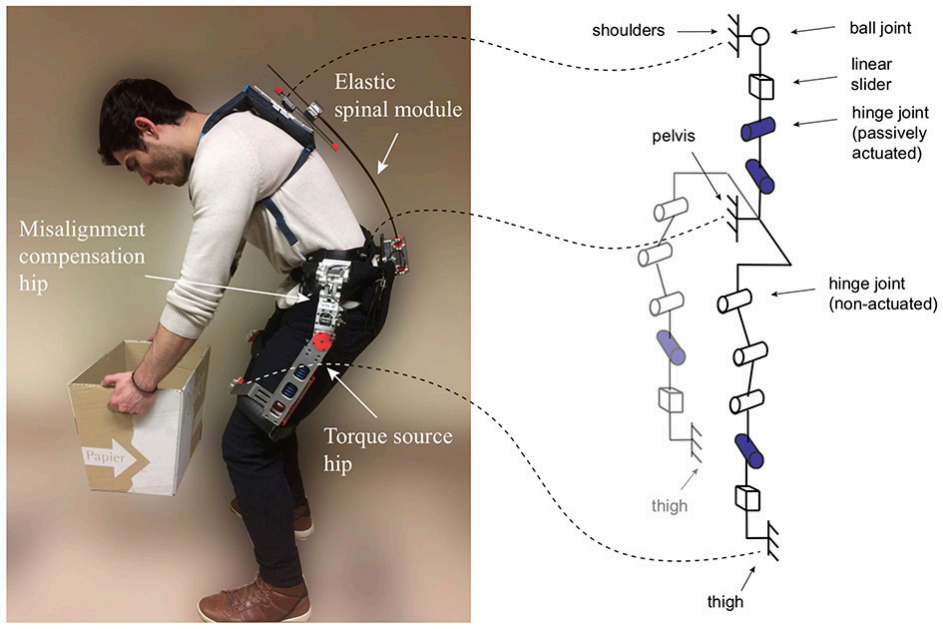
**(Left)** Passive Spexor prototype. An elastic spinal module in the back is used to provide a large range of motion and to store energy. Several misalignment compensating mechanisms at the hip and the back (hip indicated in the figure) are implemented. A passive, parallel elastic torque source provides support at the hip; **(Right)** Kinematic equivalent representation. The spinal structure is comprised of a ball joint and a linear slider in combination with elastic beams (here represented as two hinge joints in series). This structure provides flexibility and compensates for potential misalignment. The hip structure consists of three parallel hinge joints in combination with a fourth, actuated hinge joint in series with a linear joint. This structure fulfills a dual function, once as a fitting mechanism and once as a misalignment compensating structure. Written informed consent was obtained from the participant to publish this picture.

A flexible structure is used as a compensatory joint and energy storage a the same time. A combination with a linear joint along the flexible structure and an additional spherical joint was found to compensate for a large part of the misalignments in an iterative approach (See Figure 4).

#### 2.2.2. Hip structure concept

For the flexion and extension of the hip joint, a mechanism with many adjustment possibilities was chosen. Simple models form the literature indicate (Toxiri et al., 2016), that the required torque for the hip joint has a sinusoidal profile. The goal was to replicate this torque profile with adjustment possibilities (See Figure 3B) in the torque magnitude (Vanderborght et al., 2011).

Alignment of the rotation axis of a human and an exoskeleton is challenging (Junius et al., 2017a). For the flexion and extension and abduction and abduction, an approach was chosen, where a certain amount of misalignment is accepted and compensated for by the device kinematics (See Figure 2E and for more detail Figure S1), i.e., the rotational joints and the slider along the leg move to accommodate for the misalignment. Additionally, a wide range of sizes should be fitted. Based on a simple kinematic model of the human hip and the exoskeleton the workspace in the frontal plane is estimated. Anthropomorphic data from (Tilley, 2002) and range of motion estimates from (Magee, 2006) are used to estimate the human workspace (See Figure 2F and for more detail Figure S2). The kinematic equations are provided in the Supplementary Material.

Previous research on misalignment compensation around the internal/external degree of freedom of the hip indicated, that preventing the thigh cuffs from rotating around the thigh is challenging (Junius et al., 2017a). Especially, when additional compensatory degrees of freedom are introduced. The kinematic structure of the here presented exoskeleton allows for no rotation in the transverse plane, meaning the internal/external degree of freedom is blocked. However, because it is challenging to connect to the thigh in a way, that no rotation around the thigh axis occurs, we decided to use this to our advantage and implement the compensation of the internal and external rotation of the hip this way: the exoskeleton structure stays rigid and the leg rotates inside the not too much tightened cuff. The presence of large muscle groups and compliant tissues of the leg further simplify this approach.

**Figure 5:**
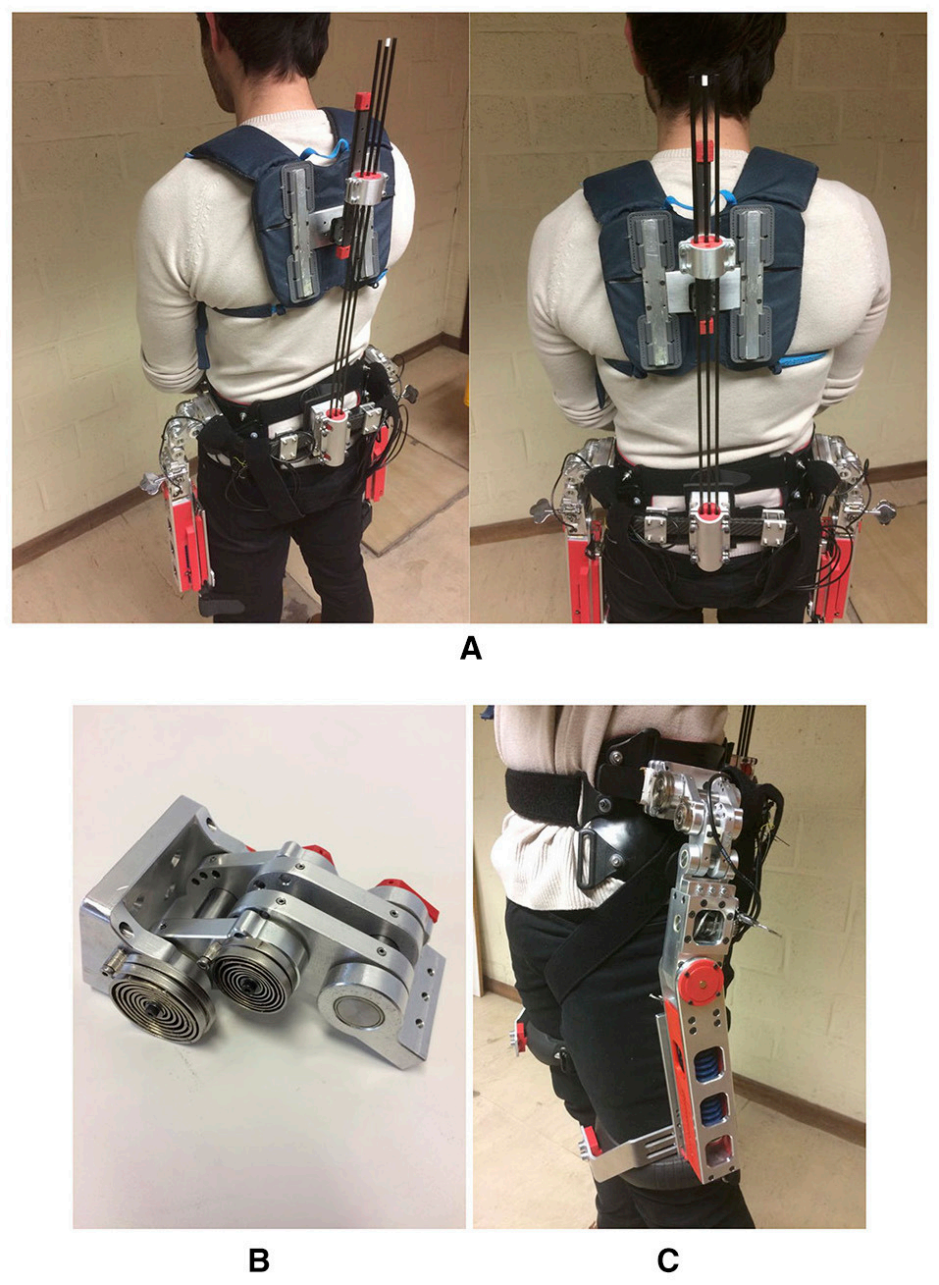
Elastic spinal module **(A)**. Three carbon fiber beams (*D* = 4 mm) provide the necessary flexibility to allow for the required range of motion, while providing a restoring torque when bent; Three parallel hinge joints **(B)**. This mechanism is on the one hand used to compensate for misalignment and on the other hand to provide a good fit. Torsional springs are mounted on the top two joints to avoid singularities and increase comfort; Misalignment compensation mechanism hip and torque source hip **(C)**. The torque, that is generated by the actuator is transmitted through the misalignment compensation mechanism.

Next to the problem to compensate for misalignment, the fitting of the exoskeleton on the hip is essential, especially, if the exoskeleton should not be protruding. One mechanism, that can be used to accommodate a wide range of different waist to hip widths, is one that consists of three parallel joints in series (See Figure 5B). Incidentally, this mechanism can also be used to compensate for a small amount of misalignment.

In the design of a controlled-brake orthosis (Durfee and Goldfarb, 1995) and later in the Robomate (Toxiri et al., 2016), a similar structure is used. The difference to our design is though, that in our case, the three compensatory joints are placed above the flexion-extension joint (See Figure 4). This allows us to use the mechanism additionally for fitting purposes and place the connection to the pelvis very close to the body. Further, this configuration avoids collisions of the exoskeleton with the leg, which could potentially occur for large abduction angles in the controlle-brake orthosis or Robomate exoskeleton.

However, this approach also comes with potential disadvantages. By placing the typically actuated and somewhat heavy flexion–extension joint below the three hinge joints for misalignment, the weight of the structure tends to rest against the skin of the wearer. Exerting a compression force at the hip. This might become a source of discomfort, especially if the actuator is heavy.

Therefore, in the presented design, the two top hinge joints are equipped with torsional springs (See Figure 5B). This prevents the relatively heavy flexion/extension joint from pressing against the hip. Additionally, the two spring loaded joints counteract the tendency of the entire mechanism, to go into a singular configuration along the leg.

In order to extend the range of motion even further, especially for lateral bending and axial rotations of the trunk, a spring loaded slider along the leg is added (See Figure 4).

The three hinge joints in combination with the linear joint, leaves the flexion - extension joint floating on both sides (“dual floating”). Potentially, this allows the flexion–extension joint to self align.

### 2.3. Mechanical implementation

In the following, the design considerations of the passive Spexor exoskeleton (See Figure 4) are described in more detail. Specifically, the physical interfaces, the spinal module, the misalignment compensation mechanisms, and the torque source at the hip.

#### 2.3.1. Physical interfaces

The pelvis structure, which connects the hip and the spinal part of the exoskeleton, is a custom made carbon fiber frame by Otto Bock HealthCare GmbH, consisting of two separate L-shaped structures that are clamped in the back. The pelvis structure is therefore adjustable in width. The mechanical loading of the pelvis structure is considerable. In order to prevent too large torsion angles of the structure, additional clamping of the ends of the overlapping structures was added (See Figure 5A).

The shoulder interface stems from a modified backpack (Karrimor Panther 65). An aluminum structure was added to mount the ball joint and to prevent the backpack from introducing unwanted slack into the system. The thigh interfaces are modified orthotics parts from Otto Bock HealthCare GMBH.

#### 2.3.2. Spinal module

In order to achieve a spinal range of motion of up to 60° in the sagittal plane, several different mechanisms were considered. Attempts to scale a 3D-printed multi-segmental prototype to torques of 20–30 Nm proved difficult due to high friction losses (Näf et al., 2017). Therefore, a new concept consisting of multiple continuous carbon fiber beams under bending loads was developed (See Figure 5A).

Advantages of the continuous beams include, that the overall structure is light weight, compact and comparatively simple. However, one of the main disadvantages is, that excessive loading breaks the beams.

The peak stresses occur at the base of the beam, therefore special attention was payed to the fixation at the base. The beams are clamped in a structure made out of 3D printed ABS plastic embedded in an aluminum structure. At the base of the structure, the ABS plastic is modeled in such a way that the plastic follows the bending curve of the beam.

The active length of the beam can be individually adjusted to the size of the user. The beams are cut to a length of 600 mm and are 4 mm in diameter. Three beams in parallel are used to provide a torque in the range of 30 Nm (See Figure 5A).

In order to dimension the beams, linear and non-linear models were used. The linear model follows a text book approach and is described in the following. Readers interested in the non-linear models are referred to Beléndez et al. (2002).

In order to calculate the displacement *v*(*x*) as a function of *x* along a cantilever beam (See Figure 3A) the following second order differential equation has to be solved:

(1) $$\begin{array}{r} \frac{d^{2}v\left(x\right)}{dx^{2}}=\frac{M}{E\cdot I} \end{array}$$

with the displacement *v*(*x*), the moment *M*, the Young's modulus *E*, and the second moment of area *I*. The Young's modulus *E* is a material property, related to stiffness, while the second moment of area *I* is related to the geometric properties. For a round cross section beam, like the one used here, the second moment of area amounts to:

(2) $$\begin{array}{r} I=\frac{\pi }{2}\cdot r^{4} \end{array}$$

with the radius *r* of the beam.

For a cantilever beam, where a force *F* is applied at the top of the beam, the moment along the beam has the following form:

(3) $$\begin{array}{r} M\left(x\right)=F\cdot \left(L-x\right) \end{array}$$

with the force *F*, the length of the beam *L* and the coordinate *x*.

This results in the following equation for the displacement *v*:

(4) $$\begin{array}{r} \frac{d^{2}v\left(x\right)}{dx^{2}}=\frac{F}{E\cdot I}\cdot \left(L-x\right) \end{array}$$

Integrating this equation twice, with the boundary conditions, that (a) the displacement at the base is zero, i.e., *v*(*x* = 0) = 0 and (b) that the deflection at the base is zero, i.e., $\frac{dv\left(x=0\right)}{dx}=0$ yields:

(5) $$\begin{array}{r} v\left(x\right)=\frac{F}{E\cdot I}\left(L\cdot x^{2}-\frac{x^{3}}{6}\right) \end{array}$$

This equation can be used to explicitly calculate the displacement *v*(*x*) as a function of the force *F*, the Young's modulus *E*, the second moment of area *I* the length *L* and the position *x* along the beam. In a similar fashion can the angle θ(*x*), which is defined as the as the difference of the displacements $\theta \left(x\right)=\frac{dv\left(x\right)}{dx}$ be calculated:

(6) $$\begin{array}{r} \theta \left(x\right)=\frac{F}{E\cdot I}\left(L\cdot x-\frac{x^{2}}{2}\right) \end{array}$$

This allows to calculate the bending angle θ(*x*) as a function of the force *F*, the Young's modulus *E*, the second moment of area *I* the length *L* and the position *x* along the beam (See Figure 9A).

Similarly, can this equation be used to estimate the Young's modulus *E*, when the force *F*, the angle θ(*x*), the second moment of area *I* and the position along the beam *x* and the overall length of the beam *L* is known.

(7) $$\begin{array}{r} E=\frac{F}{\theta \left(x\right)\cdot I}\left(L\cdot x-\frac{x^{2}}{2}\right) \end{array}$$

Qualitatively, the linear and non-linear displacements look similar. However, for large displacements, additionally a displacement δ_*x*_ along x takes place (See Figure 3A), which is not captured by the linearized equation.

#### 2.3.3. Misalignment compensating and fitting module hip

The misalignment compensation and fitting mechanism is manufactured out of aluminum (See **Figure 5B**). The distance between two parallel steel axis is 30 mm. Two parallel custom designed torsion springs are mounted on the first two joints. For future testing, the first two joints can be locked with steel pins in discrete positions, additionally, encoder mounts are foreseen on all joints.

#### 2.3.4. Torque source hip

Inverse pendulum models of the trunk predict a torque profile required around the hip to have a sinusoidal profile. In order to deliver such a torque profile, a force generated with a linear die compression spring (Sodemann ST52890), with a spring constant of 21 $\frac{\text{N}}{\text{mm}}$, is routed over a profile disk to generate a non-linear profile with two 2 mm Dyneema cables (See Figure 5C). The design is a purely passive version of a Mechanically Adjustable Compliance and Controllable Equilibrium Position Actuator (MACCEPA 2.0) as described by (Vanderborght et al., 2011). The actuator is designed in such a way, that by changing manually the pretension, the peak output torque can be adjusted in a range of 10–30 Nm per joint, amounting to a total of 20–60 Nm.

## 3. Experiments

The exoskeleton is tested in two ways: the isolated components, and the interaction of the exoskeleton with the user. The user testing is comprised of two parts, biomechanical and functional testing.

### 3.1. Component testing

In order to asses the behavior of the individual components of the exoskeleton the carbon fiber beam and the torque source at the hip were benchmarked with respect to their torque angle characteristic. This allows to estimate the torque provided by the exoskeleton with kinematic data.

**Figure 6:**
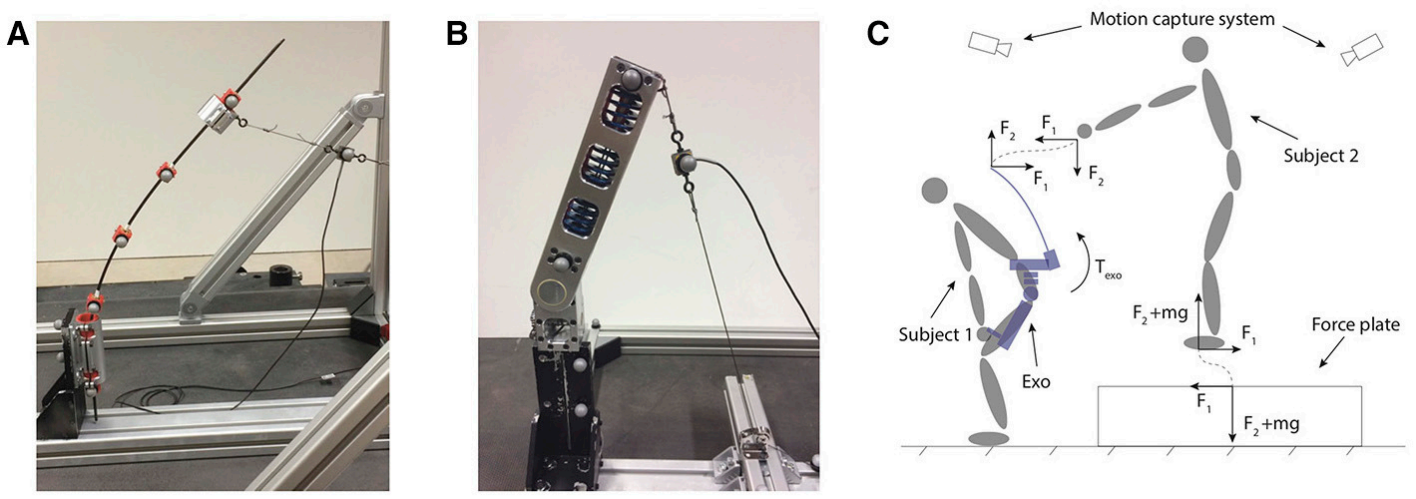
Component testing: Spinal support **(A)**. The beams are tested on the one hand to identify the material properties, such as the Young's modulus *E* and on the other hand to identify the torque angle characteristic. The carbon fiber beams are fixed on the base. A force, which is recorded with a load cell, is applied at the top. Five points on the beams are tracked using a motion capture system (Vicon Vero). Torque source hip **(B)**. In order to validate the design of the torque source and to identify the torque angle characteristic, the hip actuator is subjected to a external force. The force is recorded with a load cell, while at the same time the two markers on the rotating, top part of the actuator are tracked; Loading support verification **(C)**. Subject 1 is wearing the bottom part of the exoskeleton. The exoskeleton is equipped with markers, whose position is recorded with the motion capture system. Subject 2, with known mass *m* is standing on a force plate, while exciting the spinal part of the exoskeleton with the forces *F*_1_ and *F*_2_. The known length of the spinal part of the exoskeleton allows to calculate the support torque at lumbar level τ_exo_.

In order to benchmark the components, they were fixed in a custom made test stand (See Figure 6A,B). The components were equipped with reflective markers and their position was tracked with a camera system (Vicon Vero) at a frequency of 100 Hz. External forces, recorded with a load cell (Futek LSB 200, 445 N) at 1 kHz, were applied manually to excite the components. The recorded data was processed with custom made MATLAB scrips. The results are reported in section 4.1.

**Figure 7:**
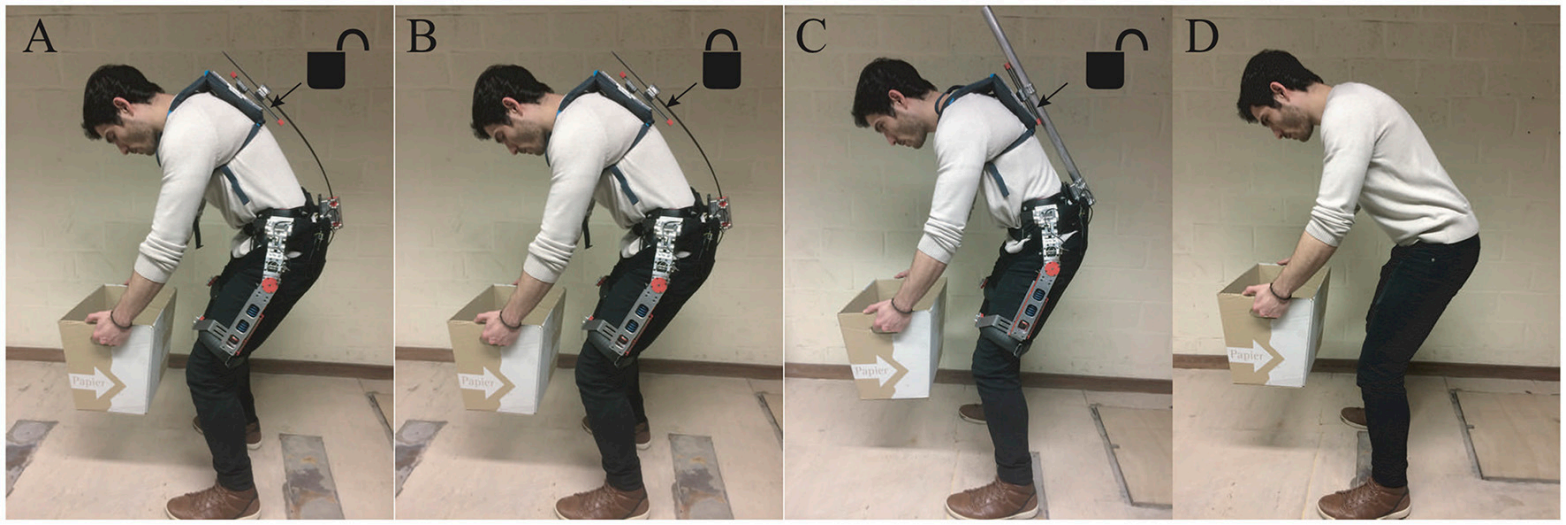
Different exoskeleton configurations were tested: **(A)** Flexible exoskeleton with slider unlocked. **(B)** Flexible exoskeleton with slider locked. **(C)** Rigid exoskeleton with slider unlocked. **(D)** No exoskeleton. Written informed consent was obtained from the participant to publish this picture.

In order to verify the loading support of the single components combined, the torque-angle behavior of the entire exoskeleton was identified. The exoskeleton was equipped with Optotrak markers, while one user wore the hip part of the exoskeleton (See Figure 6C). Instead of connecting the shoulder interface to the user, a second person standing on a force plate would excite the exoskeleton while the wearer flexes out of the way. The experiment was once performed with the flexible beams (See Figure 7A) and once with the rigid aluminum tube (See Figure 7C). The recorded data was processed with a custom MATLAB script. The resulting support torque-angle characteristic is reported in section 4.1.

### 3.2. User evaluation

All experiments involving human subjects were approved by the medical ethical committee of the VU medical center (VUmc, Amsterdam, The Netherlands, NL57404.029.16).

#### 3.2.1. Biomechanical testing

Three young, healthy male subjects (average age: 30 years, average height: 171.5 cm, average weight: 66 kg) participated in the biomechanical testing of the prototype. During the experiment, participants were asked to perform a range of motion (ROM) in all three directions; flexion-extension (y), lateral bending (x), and rotation (z), while holding the knees as straight as possible without locking the knee. Subjects were asked to put the same amount of effort in each trial when wearing the different exoskeleton configurations (See Figure 7). In total, subjects performed four maximal ROM tasks, differing in the configuration of the exoskeleton:

1. **Exo flex-slider:** Flexible back structure, with back slider unlocked
2. **Exo flex-no slider:** Flexible back structure, with back slider locked
3. **Exo rigid:** Rigid back structure, slider unlocked
4. **No Exo:** Not wearing any exoskeleton

**Figure 8:**
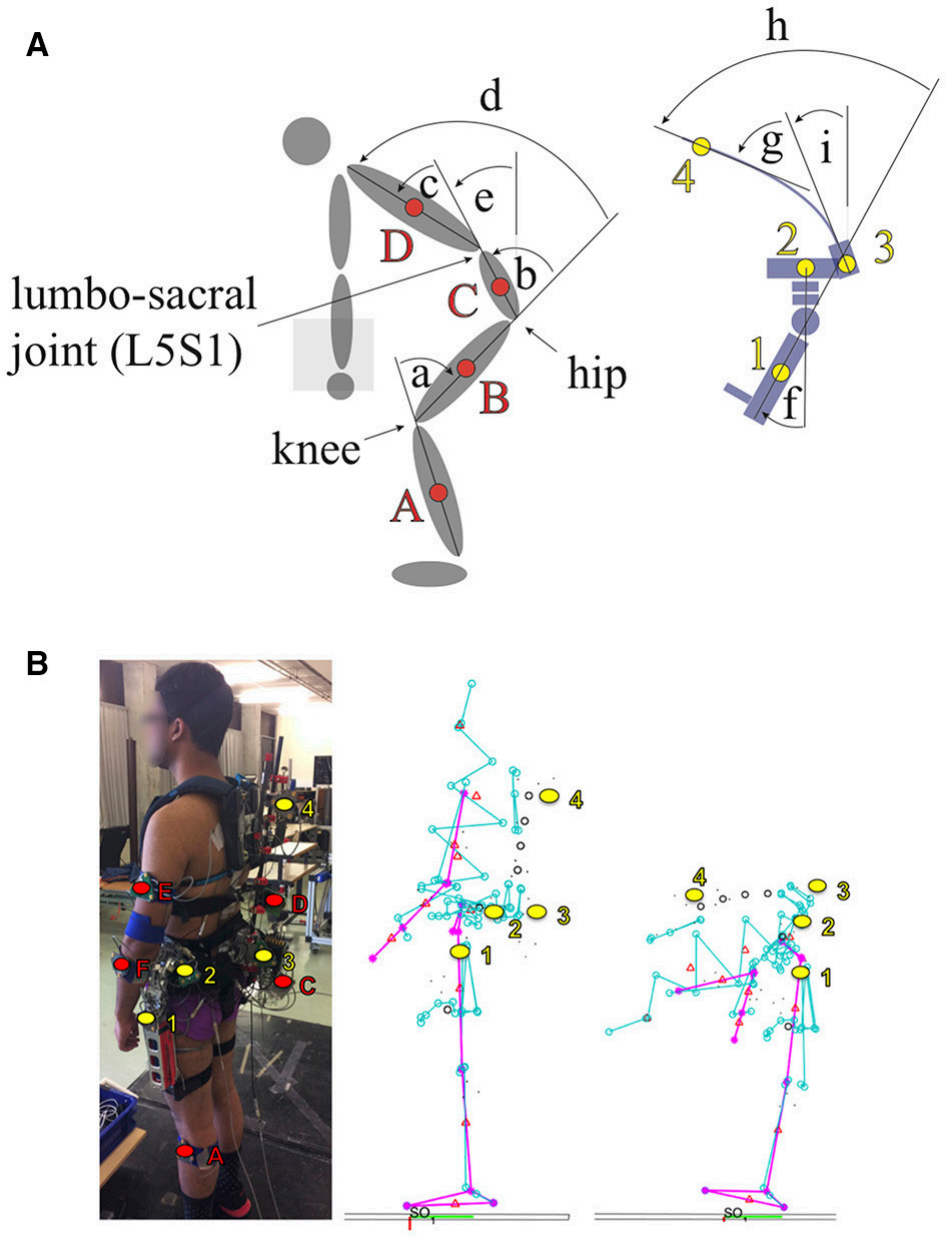
Angle definitions for the human and the exoskeleton **(A)**. Knee angle (a), hip angle (b), lumbar angle (c), trunk angle (d), pelvis inclination (e), exo hip angle (f), exo lumbar angle (g), exo trunk angle (h), exo pelvis inclination (i), Measurement setup **(B)**. Subject equipped with the exoskeleton and markers to track the position and orientation of different body parts (red). The position and orientation of the lower leg (A), upper leg (B, not visible in this photograph), the pelvis (C), the trunk (D) the upper arm (E), and the lower arm (F) are tracked. Additionally, the exoskeleton is equipped with marker clusters to track the exoskeleton (yellow) hip angle (1) the pelvis carbon fiber frame at the hip (2) the pelvis carbon fiber frame at the base of the spinal exoskeleton structure (3) top of the spinal exoskeleton structure (4). Various single markers were used to track individual parts of the exoskeleton, but are not further used in the analysis and therefore not further elaborated. The stick figures show a visualization of the tracking results. For the sake of clarity, the body markers (red) are not highlighted in this figures. In the middle the subject is standing upright. The pink lines represent body parts of the subject, while the turquoise color represents the exoskeleton structure. On the right, the subject is performing a stoop. Written informed consent was obtained from the participant to publish this picture.

3D Kinematics of both the subject and exoskeleton were measured with an optical motion capture system (Certus Optotrak, Northern Digital, Waterloo ON, Canada) at 50 Hz. Marker clusters were attached on one side of the body, to the shank (A), thigh (B), pelvis (C), thorax (D), upper arm (E), and forearm (F) (See Figure 8B). In addition, marker clusters were attached to relevant parts of the exoskeleton; exo hip joint (1), exo pelvis frame (2), exo base spinal structure (3), and exo top spinal structure (4). For all modeled human body segments (foot, shank, thigh, pelvis, abdomen, thorax, head, upper arm, forearm, and hand), anatomical coordinate systems were calculated based on anatomical landmarks that were related to the corresponding marker clusters using a probe with six markers (Cappozzo et al., 1995).

From the rotation matrices of the segments, joint angles were calculated using Euler decomposition (YXZ). The angles that were used in the analysis are defined in Figure 8A. From the angle time-series (See Figure 10), peak angles were subtracted and compared between conditions.

#### 3.2.2. Functional testing

Three men with no low-back pain history participated in the functional testing of the protoytpe. Participants performed a series of functional tasks, including (1) tasks to assess the supportive function of the device, (2) tasks to assess the extent of restricted hip flexion, and (3) tasks to asses the ROM. A detailed description of the tasks can be found in a previous study of Baltrusch et al. (2017), who developed a test battery to assess the effect of a passive trunk exoskeleton on functional performance. For convenience the tasks are also listed in Figure 12B.

The functional tasks were performed once with an exoskeleton and without any exoskeleton. The sequence of the tasks and the starting conditions were randomized to prevent order and habituation effects. Functional performance was assessed by using questionnaires after each task, asking for perceived task difficulty and discomfort of the device. At the end of the session participants had to fill in a user's impression questionnaire regarding their experience with the exoskeleton during the test session. The subjective outcomes measures were all assessed by using a visual-analog scale (VAS). This allows to more accurately distinguish between participants opinion compared to numeric scales (Kersten et al., 2012).

*User's impression*: The users impression questionnaire was divided into the assessment of range of motion, efficacy, and overall impression of the prototype. For range of motion participants got asked “Are you restricted in your freedom of movement?” with a VAS scale ranging from “not restricted” to “heavily restricted.” For assessing the efficacy of the device participants had to answer questions based on the reduction of back loading (“Does the device reduce the loading on your back?”; 0 = high reduction, 10 = no reduction), the support of tasks (“Does the device support you in performing the tasks you did?”; 0 = high support, 10 = no support) and the interference with tasks (“Does the device interfere with the tasks you did?”; 0 = no interference, 10 = high interference). To assess the overall impression participants were instructed to grade the device on a VAS scale with 0 = very bad and 10 = perfect.

*Perceived task difficulty*: To indicate the perceived task difficulty after each task participants were asked to put a cross on a VAS scale ranging from “very easy” to“very difficult,” with the question: “How difficult was the task you just performed?.” This outcome was assessed in the control and in the exoskeleton condition.

*Discomfort*: In the exoskeleton conditions participants were asked to indicate the discomfort of the prototype after each task by putting a cross on a VAS scale that ranged from “very comfortable” to “very uncomfortable.”

The results of the functional testing are presented in section4.3.

## 4. Results

In this section, first, the results of the component testing are presented, followed by the biomechanical and functional testing of the exoskeleton with users.

### 4.1. Component testing

**Figure 9:**
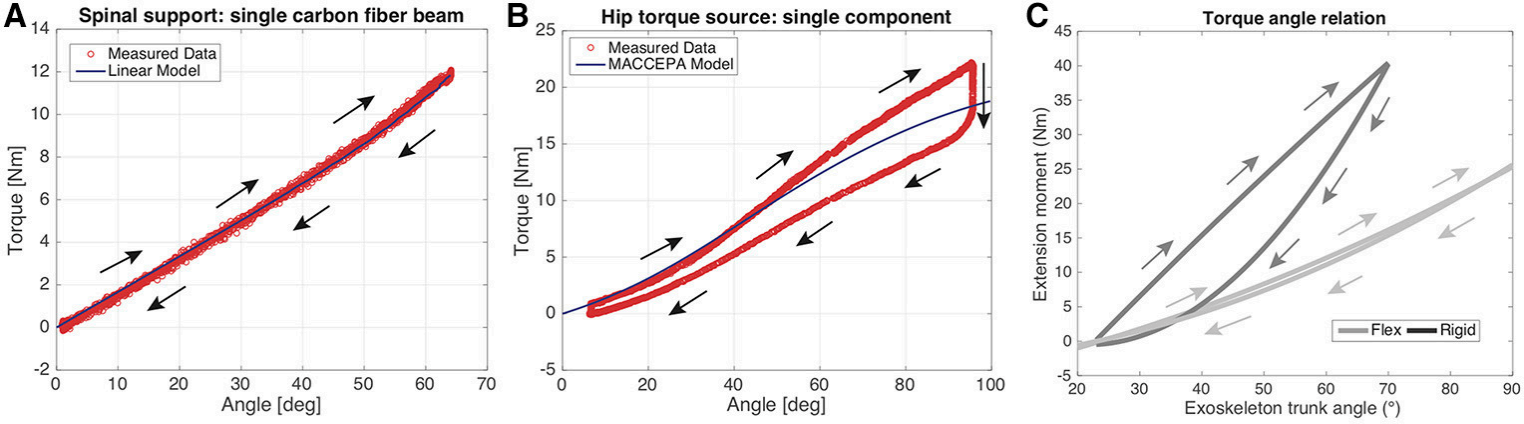
Spinal support **(A)**. Measured data of one single beam is compared to the data of the model. The model and the measurements coincide. An angular range of 64° was measured which produced a torque of 12 Nm. Torque source hip **(B)**. Measured data is compared to an analytical model of the MACCEPA 2.0. Up to an angle of 40° the two models coincide for loading. For angles bigger than 40° the measurement data exceeds the modeled torque. For an angular displacement of 95° an output torque of 22 Nm is produced. During unloading, the torque source shows hysteresis. Entire exoskeleton stiffness **(C)**: The entire exoskeleton stiffness is characterized, once with flexible beams mounted (Flex, see Figure 7A) and once with a rigid back interface (Rigid, see Figure 7C) mounted.

The results of the component testing can be found in Figure 9. One single carbon fiber beam produced an output torque of 12 Nm at an angle of 64°. Three beams in parallel, therefore produce a peak torque of 36 Nm. One hip torque source with a pretension of 30% (2.15 cm) produced a peak output torque of 22 Nm, two therefore a peak torque of 44 Nm. The entire exoskeleton consisting of flexible beams produced an output torque of 25 Nm at a trunk angle of 90°. The rigid exoskeleton produced a torque of up to 40 Nm at a trunk angle of 70°.

### 4.2. Biomechanical testing

#### 4.2.1. Kinematics

**Figure 10:**
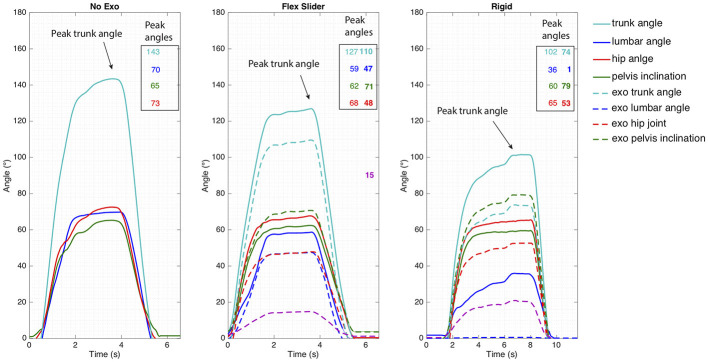
Typical time series of the range of motion measurements. Three cases are illustrated (from the left to the right): Not wearing an exoskeleton, wearing an exoskeleton with a flexible spinal interface and an unlocked spinal slider configuration and wearing an exoskeleton with a rigid spinal interface and also unlocked slider configuration. In the last two panels additionally exoskeleton angles are shown. A decrease of peak trunk flexion angles from 143° to 127° to 102° is visible for the three configurations. The movements with the rigid exoskeleton were performed slower.

**Figure 11:**
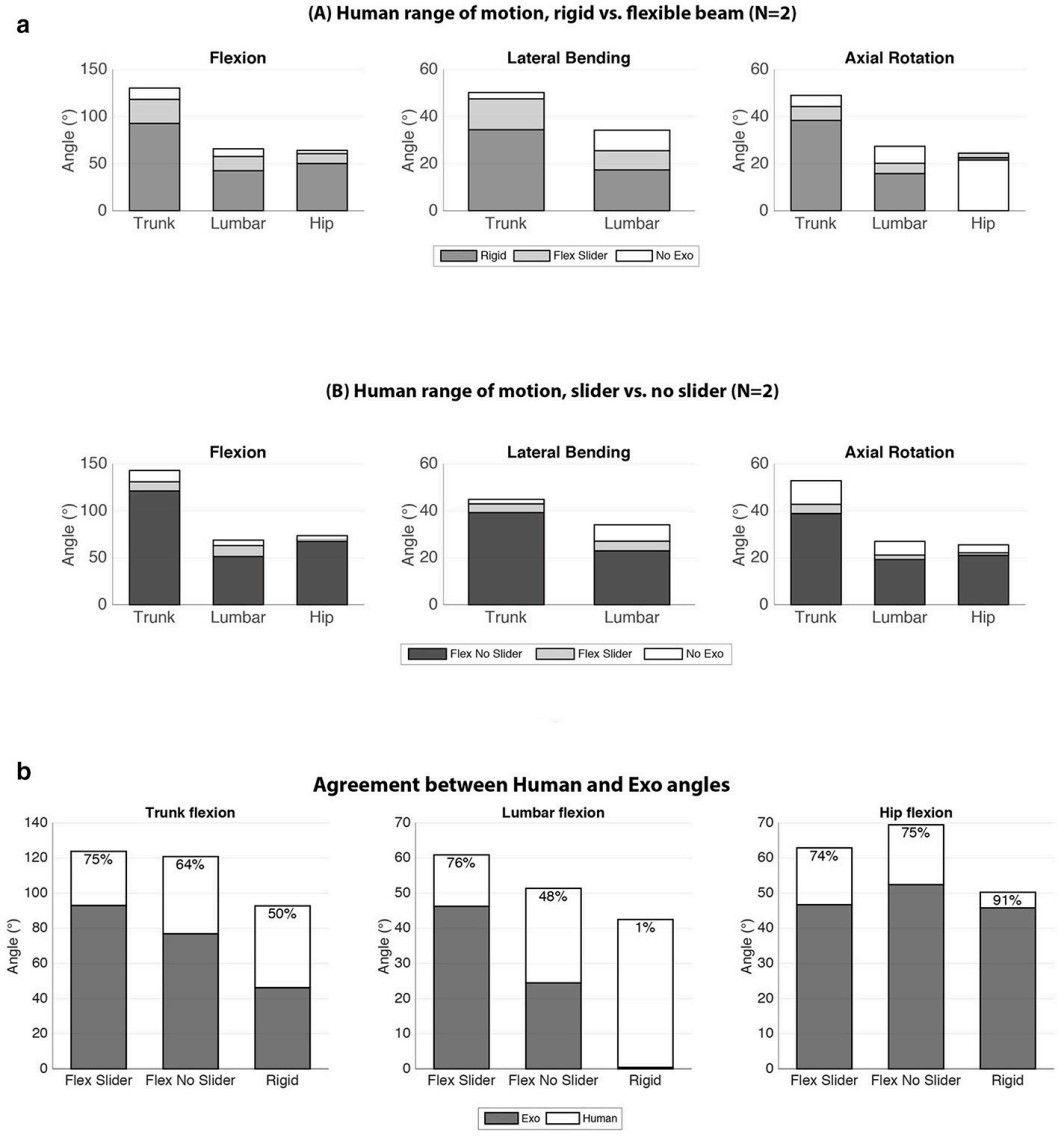
Bio-mechanical testing **(a)**: (A) The range of motion of the test subjects while: not wearing an exoskeleton (white), wearing an exoskeleton with flexible beams as spinal interface (light gray), and wearing an exoskeleton with a rigid aluminum tube as spinal interface (dark gray). (B) The range of motion of the subjects while: not wearing an exoskeleton (white), wearing an exoskeleton with flexible beams as spinal interface, slider unlocked (light gray), wearing an exoskeleton with flexible beams as spinal interface, slider locked (dark gray) **(b)** Comparison between the angles measured in the subjects and the corresponding angle in the exoskeleton.

A typical time series of the range of motion testing is displayed in Figure 10. Good correspondence between the human and Exo angles were observed with correlation values above 0.98. Largest trunk flexion occurred in the No Exo condition and decreased with 16° and 41° for the Flex Slider and Rigid condition, respectively. In all cases, human peak angles while wearing the Exo decreased with respect to the No Exo condition. Bar plots indicating the peak angles of the range of motion trials and agreement between the Human and Exo peak angles are shown in Figure 11. In all three directions, peak angles were bigger in the Flex Slider condition compared to the Rigid condition. Almost no loss in ROM was found in lateral bending between No Exo and Flex Slider. However, between Rigid and Flex slider, lateral bending ROM decreased with more than 13°. As can be seen in the lower panel of Figure 11, a part of the improvement of the ROM is due to the effect of the slider and is thus not only due to the use of the flexible beams as was shown in the top panel of Figure 11. Figure 11b shows the agreement between the Human and Exo angles. In the no slider condition, agreement between Human and Exo lumbar flexion reduces with 18% compared to the agreement in the Flex Slider condition. In addition, a reduction of 47 % is observed due to the difference between Flex No Slider and Rigid. Showing the benefit of the proposed features in both absolute angles as well as following the movement of different segments of the human.

### 4.3. Functional testing

The users impression of the device as assessed in the Users Impression Questionnaire show very good results for the categories “ROM” and “Interference with tasks” and good to moderate values for the categories “Reduction of back loading,” “Support of tasks,” and “Overall impression” (See Table 2). We contrasted our results with the results of the study from Baltrusch et al. (2017), to indicate improvements in the prototype compared to the Laevo device, based on user's impressions.

**Table 2:** Users impression assessed by VAS scales: Numbers in brackets indicate the results of the study measured with Laevo in the study of Baltrusch et al. (2017).

		**Median**	**Interquartile range**	**VAS scale**
Range of motion		1.4 (4.1)	1.3–1.5 (3.1–5.9)	0 = not restricted
				10 = heavily restricted
Efficacy	Reduction of	6.1 (6.3)	3.9–6.1 (4.1–7.8)	0 = high reduction
	back loading			10 = no reduction
	Support of	5.9 (6.4)	4.1-6 (4.9–7.6)	0 = high support
	tasks			10 = no support
	Interference	1.7 (3.3)	1.6–3.1 (2.0–5.9)	0 = no interference
	with tasks			10 = high interference
Overall		7 (6)	6–8 (4–6)	0 = very bad
impression				10 = perfect

**Figure 12:**
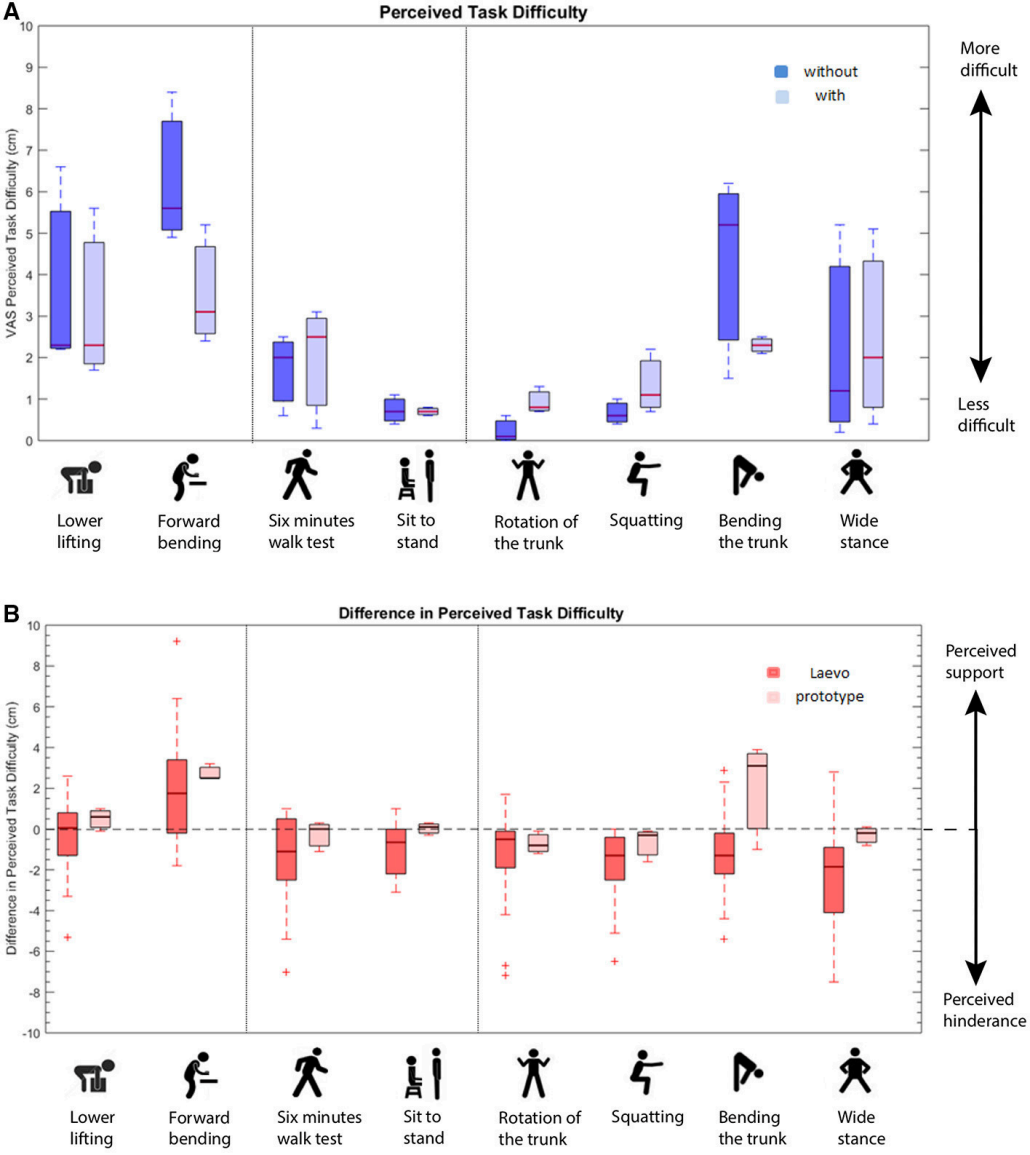
**(A)** Boxplots of perceived task difficulty. The red line represents the sample median. The distances between the tops and bottoms are the interquartile ranges. Whiskers show the min and max values; The dotted lines represent the division between (1) tasks to assess the supportive function of the device (right side), (2) tasks to assess the extent of restricted hip flexion (middle), and (3) tasks to asses the ROM (right side); 0 = very easy, 10 = very difficult. **(B)** Comparison of the Laevo device assessed in Baltrusch et al. (2017) and the prototype of the current study: Boxplots of difference in perceived task difficulty between without and with exoskeleton condition. Values above the dashed zero baseline indicate a support by the device, values below the baseline indicate a hindrance by the device. The red line represents the sample median. The distances between the tops and bottoms are the interquartile ranges. Whiskers show the min and max values; The dotted lines represent the division between 1) tasks to assess the supportive function of the device (right side), 2) tasks to assess the extent of restricted hip flexion (middle), and 3) tasks to asses the ROM (right side).

The Perceived task difficulty decreased when wearing the exoskeleton in one of the tasks to assess the supportive character of the device and did not change in the other one. Tasks to assess the extend of restricted hip flexion showed no change or a slight increase in perceived task difficulty when wearing the prototype. Participants perceived the range of motion tasks as slightly more difficult, with the exception of forward bending, which was perceived easier to perform with the exoskeleton (See Figure 12A).

We again contrasted these results to the values of Baltrusch et al. (2017) assessed when testing the Laevo device (See Figure 12B), by comparing the median change in perceived task difficulty. Hence, values above zero indicate support by the device, whereas values below zero indicate hindrance by the device when performing the task.

With regard to discomfort levels, the physical user interface shows promising results. Participants reported low discomfort, ranging from 0.6 cm on the VAS scale for wide stance up to 4.4 cm for walking. Problems that were still mentioned are touching of the leg pads during walking, friction on the side of the neck and pressure on the hips.

## 5. Discussion

A passive back support exoskeleton for industrial applications, that preserves the natural range of motion of the user was designed and tested. The exoskeleton uses misalignment compensation for both the lumbo-sacral (L5/S1) joint and the hip joint. We have demonstrated, that using flexible beams as a back interface increases the trunk range of motion by more than 25% (24.5°) compared to its rigid counterpart in the sagittal plane (See Figure 11). With the flexible beams, the range of motion is only decreased by 10% (13°) compared to not wearing an exoskeleton at all. An average range of motion of 117° in the sagittal plane was measured in the prototype with the flexible beam as spinal interface. Compared to the design goal of 60° flexion-extension in the sagittal plane of the Robomate exoskeleton (Toxiri et al., 2016), that is an increase of almost 100% (57°).

Encouraging are also the results, that an angle agreement between the exoskeleton and the human of more than 70% was achieved if the full set of misalignment compensating mechanisms is enabled (See Figure 11). Meaning, that the angles of the exoskeleton are only around 30% smaller than the corresponding angles of the human. Of special interest here is, that both the lumbar and the hip angle are 30% reduced compared to the human angles. In this case, no literature values to compare to are available. The Back Support Muscle Suit is one of the few designs that also includes a flexion-extension joint for the lumbar spine (Muramatsu et al., 2013). However, no data was published on how big the contributions of these individual angles were, while wearing the device. The angle agreement results are especially striking, if we compare these results to the case of the rigid exoskeleton (See Figure 11) : The agreement between the lumbar angles is as low as one percent. Meaning, that the exoskeleton structure accounted for only one percent of the lumbar angle in the human. However, this discrepancy is to some degree compensated by the hip angle, which agrees by more than 90% in this case. For the overall trunk flexion however, the difference is still a 50% discrepancy between the exoskeleton trunk angles and the human trunk angle.

The flexible exoskeleton structure produces a torque of up to 25 Nm, which places it in a similar range as state of the art passive exoskeletons such as the PLAD, which produces torque of up to 27.5 Nm. With the rigid aluminum tube as spinal structure, a torque of up to 40 Nm can be produced, however, at the cost of a reduced range of motion by 24.5° and an angle discrepancy between the exoskeleton of up to 50%. Further research is needed, to investigate, if good angle agreement can also be achieved with higher support torques from a flexible structure.

A comparison to the Laevo device with functional outcomes draw an encouraging picture. Especially big is the difference when asked to rate the interference of tasks by the devices. While the interference with the described prototype is perceived as low as 1.7 cm on the VAS scale, the Laevo almost doubles this value with 3.4 cm (See Figure 12A). Note, a value of 0 cm means no interference and a value of 10 cm means a high interference.

The device is perceived to especially simplify the task of forward bending during a manual task and bending the trunk forward as much as possible, with extended knees. On the other hand walking seems to become more difficult. This is not surprising, because in a passive device, the user has to work against the device while pushing the leg forward. A clutch mechanism, that engages the hip torque source during a lifting task and disengages is during walking, stair climbing etc. is already under development. A purely passive clutch mechanism of this kind has been integrated in the BackX exoskeleton. However, no studies or data could be found, that investigate how well this mechanism works and how its function is perceived by the user.

### 5.1. Limitations

The number of subjects for the functional tests (*N* = 3) is small, especially considering the subjective nature of these tests. This means, that generalizations of the functional testing results should be interpreted with caution. Nonetheless should be noted, that for this small group of participants consistent and perceivable improvements compared to the Laevo exoskeleton were reported by the participants.

## 6. Conclusions

A passive novel back support exoskeleton was presented, which allows for a large range of motion while wearing it. At the same time support torques of up to 25 Nm are provided at the lower back. Good kinematic agreement, resulting from the misalignment compensation mechanisms at the trunk and hip level minimize the relative movement between the exoskeleton and the user. A comparison of functional pilot test outcomes with previous results with the Laevo device suggests, that the exoskeleton is perceived less hindering in almost all tested cases.

## Author contributions

MN, CR-G, BV, and DL developed the concept. MN developed the technology with inputs from CR-G, BV, and DL. MN, AK, and SB and CR-G conceived and designed the experiments. CR-G, AK, SB, and BV contributed to the design and layout of the article, tables, additions to the bibliography, and extensive revisions. CR-G, BV, and DL helped to obtain the funding for the project that financed this research.

### Conflict of interest statement

The authors declare that the research was conducted in the absence of any commercial or financial relationships that could be construed as a potential conflict of interest.

## References

[B1] Abdoli-Eramaki M. Agnew M. J. Stevenson J. M. (2006). An on-body personal lift augmentation device (PLAD) reduces EMG amplitude of erector spinae during lifting tasks. Clin. Biomech. 21, 456–465. 10.1016/j.clinbiomech.2005.12.021 16494978

[B2] Abdoli-Eramaki M. Stevenson J. M. Reid S. A. Bryant T. J. (2007). Mathematical and empirical proof of principle for an on-body personal lift augmentation device (PLAD). J. Biomech. 40, 1694–1700. 10.1016/j.jbiomech.2006.09.006 17466313

[B3] Babič J. Mombaur K. Lefeber D. van Dieën J. Graimann B. Russold M. (2017). Spexor: Spinal exoskeletal robot for low back pain prevention and vocational reintegration, in Wearable Robotics: Challenges and Trends, eds González-Vargas J. Ibáñez J. Contreras-Vidal J. L. van der Kooij H. Pons J. L. (Cham: Springer International Publishing), 311–315.

[B4] Baltrusch S. van Dien J. van Bennekom C. Houdijk H. (2017). The effect of a passive trunk exoskeleton on functional performance in healthy individuals. Appl. Ergon. 72, 94–106. 10.1016/j.apergo.2018.04.007 29885731

[B5] Barrett A. L. Fathallah F. A. (2001). Evaluation of four weight transfer devices for reducing loads on lower back during agricultural stoop labor, in ASAE Annual International Meeting, 2001 (Sacramento, CA).

[B6] Beléndez T. Neipp C. Beléndez A. (2002). Large and small deflections of a cantilever beam. Eur. J. Phys. 23:371 10.1088/0143-0807/23/3/317

[B7] Bosch T. van Eck J. Knitel K. de Looze M. (2016). The effects of a passive exoskeleton on muscle activity, discomfort and endurance time in forward bending work. Appl. Ergon. 54, 212–217. 10.1016/j.apergo.2015.12.003 26851481

[B8] Cappozzo A. Catan F. Crocel U. D. Leardini A. (1995). Position and orientation in space of bones during movement : anatomical frame definition and determination. Clin. Biomech. 10, 171–178. 11415549 10.1016/0268-0033(95)91394-t

[B9] Christophy M. Senan N. A. F. Lotz J. C. O'Reilly O. M. (2012). A Musculoskeletal model for the lumbar spine. Biomech. Model. Mechanobiol. 11, 19–34. 10.1007/s10237-011-0290-6 21318374

[B10] Coenen P. Gouttebarge V. van der Burght A. S. van Dieën J. H. Frings-Dresen M. H. W. van der Beek A. J.. (2014). The effect of lifting during work on low back pain: a health impact assessment based on a meta-analysis. Occup. Environ. Med. 71, 871–877. 10.1136/oemed-2014-102346. 25165395

[B11] Dagenais S. Caro J. Haldeman S. (2008). A systematic review of low back pain cost of illness studies in the United States and internationally. Spine J. 8, 8–20. 10.1016/j.spinee.2007.10.005 18164449

[B12] de Looze M. P. Bosch T. Krause F. Stadler K. S. O'Sullivan L. W. (2015). Exoskeletons for industrial application and their potential effects on physical work load. Ergonomics 59, 671–681. 10.1080/00140139.2015.1081988 26444053

[B13] Durfee W. and Goldfarb, M. (1995). Controlled-brake Orthosis. US Patent 5,476,441. Washington, DC.

[B14] Eurofound (2012). Fifth European Working Conditions Survey. Technical report, Publications Office of the European Union.

[B15] Imamura Y. Tanaka T. Suzuki Y. Takizawa K. Yamanaka M. (2014). Analysis of trunk stabilization effect by passive power-assist device. J. Robot. Mechatron. 26, 791–797. 10.20965/jrm.2014.p0791

[B16] Inose H. Mohri S. Arakawa H. Okui M. Koide K. Yamada Y. (2017). Semi-Endoskeleton-Type Waist Assist AB-Wear Suit Equipped with Compressive Force Reduction Mechanism, in 2017 IEEE International Conference on Robotics and Automation (ICRA) (Singapore), 2–7.

[B17] Junius K. Degelaen M. Lefeber N. Swinnen E. Vanderborght B. Lefeber D. (2017a). Bilateral, misalignment-compensating, full-DOF hip exoskeleton: design and kinematic validation. Appl. Bionics Biomech. 2017:5813154 10.1155/2017/5813154 28790799 PMC5534269

[B18] Junius K. Lefeber N. Swinnen E. Vanderborght B. Lefeber D. (2017b). Metabolic effects induced by a kinematically compatible hip exoskeleton during STS. IEEE Trans. Biomed. Eng. 65, 1399–1409. 10.1109/TBME.2017.2754922 28945586

[B19] Kersten P. Tennant A. Kucukdeveci A. (2012). Reply on How should we use the Visual Analogue Scale (VAS) in Rehabilitation Outcomes. Found. Rehabil. Inform. 44, 803–804. 10.2340/16501977-1044 22674245

[B20] Kingma I. Baten C. T. M. Dolan P. Toussaint H. M. Van Dieën J. H. De Looze M. P.. (2001). Lumbar loading during lifting: a comparative study of three measurement techniques. J. Electromyogr. Kinesiol. 11, 337–345. 10.1016/S1050-6411(01)00011-6 11595553

[B21] Kingma I. Faber G. S. van Dieen J. H. (2010). How to lift a box that is too large to fit between the knees. Ergonomics 53, 1228–1238. 10.1080/00140139.2010.512983 20865606

[B22] Laevo B. V. (2018). Product Information - What is the Laevo and How Does It Work?. Available online at: http://en.laevo.nl (Accessed January, 2018).

[B23] Lavender S. A. Ko P. L. Sommerich C. M. (2013). Biomechanical evaluation of the Eco-Pick lift assist: a device designed to facilitate product selection tasks in distribution centers. Appl. Ergon. 44, 230–236. 10.1016/j.apergo.2012.07.006 22884290

[B24] Magee D. J. (2006). Orthopedic Physical Assessment, 4th Edn Philadelphia, PA: Elsevier Health Sciences.

[B25] Millard M. Sreenivasa M. Mombaur K. (2017). Predicting the motions and forces of wearable robotic systems using optimal control. Front. Robot. AI 4:41 10.3389/frobt.2017.00041

[B26] Muramatsu Y. Umehara H. Kobayashi H. (2013). Improvement and quantitative performance estimation of the back support muscle suit, in Engineering in Medicine and Biology Society (EMBC), 2013 35th Annual International Conference of the IEEE (Osaka: IEEE), 2844–2849. 10.1109/EMBC.2013.6610133 24110320

[B27] Näf M. B. De Rijcke L. Guerrero C. R. Millard M. Vanderborght B. Lefeber D. (2017). Towards low back support with a passive biomimetic exo-spine, in 2017 International Conference on Rehabilitation Robotics (ICORR) (London: IEEE), 1165–1170. 10.1109/ICORR.2017.8009407 28813979

[B28] Netter F. H. (2011). Atlas of Human Anatomy, 5th Edn Philadelphia, PA: Saunders Elsevier.

[B29] Pons J. L. (2008). Wearable Robots: Biomechatronic Exoskeletons. Chichester: John Wiley & Sons.

[B30] Schiele A. (2009). Ergonomics of exoskeletons: subjective performance metrics, in 2009 IEEE/RSJ International Conference on Intelligent Robots and Systems, IROS 2009 (St. Louis, MO), 480–485.

[B31] Schiele A. van der Helm F. C. (2006). Kinematic design to improve ergonomics in human machine interaction. IEEE Trans. Neural Syst. Rehabil. Eng. 14, 456–469. 10.1109/TNSRE.2006.881565 17190037

[B32] Schorsch J. Keemink A. Q. L. Stienen A. Van der Helm F. Abbink D. (2014). A novel self-aligning mechanism to decouple force and torques for a planar exoskeleton joint. Mech. Sci. 5:29 10.5194/ms-5-29-2014

[B33] SuitX (2018). backX. Available online at: http://www.suitx.com/backx (Accessed January, 2018).

[B34] Tilley A. R. (2002). The Measure of Man and Woman: Human Factors in Design, Vol. 1 New York, NY: John Wiley & Sons.

[B35] Toxiri S. Ortiz J. Masood J. Fernandez J. Mateos L. A. Caldwell D. G. (2016). A wearable device for reducing spinal loads during lifting tasks: biomechanics and design concepts, in 2015 IEEE International Conference on Robotics and Biomimetics, IEEE-ROBIO 2015 (Zhuhai), 2295–2300.

[B36] Ulrey B. L. and Fathallah, F. A. (2013). Effect of a personal weight transfer device on muscle activities and joint flexions in the stooped posture. J. Electromyogr. Kinesiol. 23, 195–205. 10.1016/j.jelekin.2012.08.014 23021604

[B37] Vanderborght B. Tsagarakis N. G. Van Ham R. Thorson I. Caldwell D. G. (2011). Maccepa 2.0: compliant actuator used for energy efficient hopping robot chobino1d. Auton. Robots 31, 55–65. 10.1007/s10514-011-9230-7

[B38] Waters T. R. Putz-Anderson V. Garg A. (1994). Applications Manual for the Revised NIOSH Lifting Equation. Cincinnati, OH: Center for Disease Control and Prevention.

[B39] Waters T. R. Putz-Anderson V. Garg A. Fine L. J. (1993). Revised NIOSH equation for the design and evaluation of manual lifting tasks. Ergonomics 36, 749–776. 8339717 10.1080/00140139308967940

[B40] Wehner M. Rempel D. Kazerooni H. (2009). Lower extremity exoskeleton reduces Back forces in lifting, in ASME 2009 Dynamic Systems and Control Conference, Vol. 2 (Hollywood, CA), 49–56.

[B41] Zhang H. Kadrolkar A. Sup F. C. (2016). Design and preliminary evaluation of a passive spine exoskeleton. J. Med. Devices 10, 10–17. 10.1115/1.4031798

