# Supplementary material for: Passive Back Support Exoskeleton Improves Range of Motion Using Flexible Beams
Source: Front Robot AI. 2018 Jun 21;5:72. doi: 10.3389/frobt.2018.00072 (PMC7805753; doi:10.3389/frobt.2018.00072)
Supplement: Supplementary file 1 [file Presentation_1.PDF]

Supplementary Material: Passive Back Support Exoskeleton Improves Range of Motion Using Flexible Beams

1 SUPPLEMENTARY DATA

1.1 Kinematic equations

For visualisation purposes, the workspace of the human hip joint and the exoskeleton are calculated in the frontal plane. In the following, the kinematic equations modelling a human hip joint in the frontal plane are presented (See Figure S1). Further, a kinematic model of the hip misalignment compensation of the exoskeleton are introduced.

1.1.1 Human kinematic model

The hip joint is modelled as a ball joint. The modelling is done with respect to the coordinate system X_0, Y_0. The equation for the mid point of the femur, which was chosen as the point where the exoskeleton should interact, is found as:

$$X_I = -lx_H + r_I \cdot \sin(\theta) \tag{S1}$$

$$Y_I = -ly_H + r_I \cdot \cos(\theta) \tag{S2}$$

where lx_H and ly_H denote an approximations of the distance from the iliac crest to the hip joint center (estimated with data from (Tilley, 2002)). Here, an average of the two extreme cases: 1 percentile man and 99 percentile woman is taken, which are the extreme cases for this specific measure. Leading to the distances $lx_H = 58$ mm and $ly_H = 114$ mm. For the distance r_I from the hip joint center to the middle of the femur, the extreme cases are the 99 percentile man and the 1 percentile woman. r_I is the linear interpolation between these cases $r_I = r_{Imin} + (r_{Imax} + r_{Imin}) \cdot i/N$ with $i = \{1, N\}$, $N = 100$. According to (Tilley, 2002) $r_{Imin} = 175.5$ mm and $r_{Imax} = 233.5$ mm. For the human hip angle θ in the frontal plane, the range $\theta = \{-30°, 50°\}$ according to (Magee, 2006) is considered.

1.1.2 Exoskeleton kinematic model

In a similar fashion the the human kinematic model, the exoskeleton is modelled with respect to the coordinate system X_0, Y_0. The equation for the kinematic chain is found to be:

$$X_I = r_0 + r_1 \cdot \cos(\phi_1) + r_2 \cdot \cos(\phi_1 + \phi_2) + (l_1 + l) \cdot \cos(\phi_1 + \phi_2 + \phi_3) + l_2 \cdot \cos(\phi_1 + \phi_2 + \phi_3 + \phi_{40}) \tag{S3}$$

$$Y_I = -r_1 \cdot \sin(\phi_1) - r_2 \cdot \sin(\phi_1 + \phi_2) - (l_1 + l) \cdot \sin(\phi_1 + \phi_2 + \phi_3) - l_2 \cdot \sin(\phi_1 + \phi_2 + \phi_3 + \phi_{40}) \tag{S4}$$

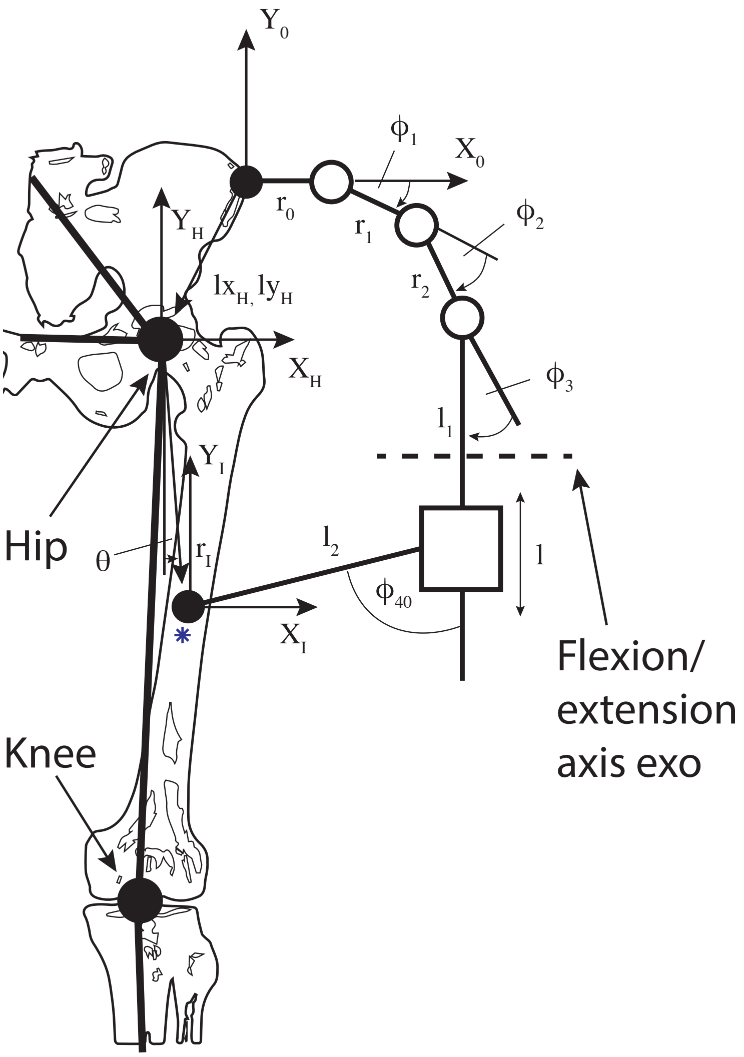

Figure S1: Definition of coordinate systems, angles and length for the kinematic model

with the distance r_0, that the first joint is away from the iliac crest (r_0 = 47.4 mm), the link length $r_1 = r_2 =$ 30 mm, $l_1 =$ 130 mm and l_2 = 168 mm. No effects from different planes are considered in this model. For instance the effect of flexion/extension on the link length l_1 is not considered. When the joints are unlocked, the angles ϕ_1, ϕ_2 and ϕ_3 are variable, while $\phi_{40} = 53°$ is constant. The following ranges are considered for the the analysis: $\phi_1 = \{-50°, 90°\}$, $\phi_2 = \{-45°, 45°\}$, $\phi_3 = \{-80°, 90°\}$ and $l = \{0 \text{ mm}, 100 \text{ mm}\}$

The resulting workspace of both is visualized in Figure S2.

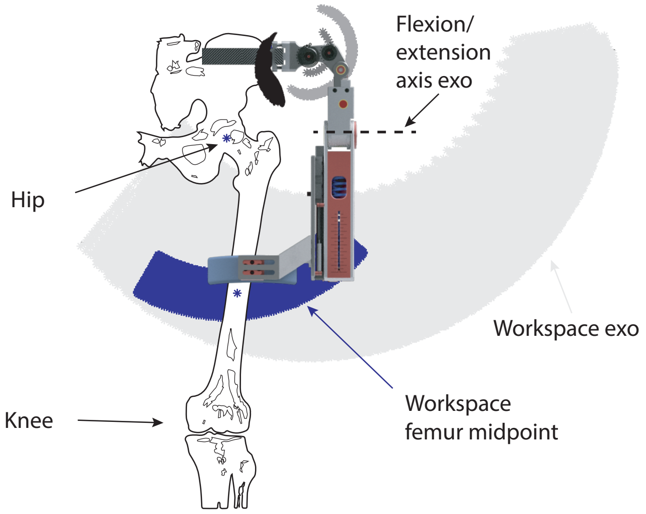

Figure S2: Comparison between the hip workspace in the frontal plane of the human hip and the exoskeleton

REFERENCES

Magee, D. J. (2006). *Orthopedic Physical Assessment* (Elsevier Health Sciences), 4 edn.

Tilley, A. R. (2002). *The measure of man and woman: human factors in design*, vol. 1 (John Wiley & Sons)
